# Primary Vaginal Neuroendocrine Carcinoma: Case Report and Literature Review

**DOI:** 10.7759/cureus.102419

**Published:** 2026-01-27

**Authors:** Yanina Nikolaus, Saroj Sigdel

**Affiliations:** 1 Pathology, Marshall University Joan C. Edwards School of Medicine, Huntington, USA

**Keywords:** atezolizumab, case report, extrapulmonary small cell carcinoma, high-grade neuroendocrine tumor, hpv-associated gynecologic malignancy, immunotherapy, insm1 immunohistochemistry, platinum–etoposide chemotherapy, primary small cell carcinoma of the vagina, vaginal neuroendocrine carcinoma

## Abstract

Primary small cell carcinoma of the vagina (SmCCV) is exceptionally rare, and its presentation often mimics metastatic disease from more common sites, making diagnosis difficult. In this report, we describe an older woman who came to medical attention with new vaginal bleeding, leading to the discovery of a large vaginal mass. Biopsy findings and whole-body imaging, including positron emission tomography/computed tomography (PET/CT), ultimately supported a primary vaginal origin despite the presence of extensive metastases. Her case illustrates how unusual and clinically challenging this tumor can be, and it emphasizes the need for close collaboration between clinical evaluation, imaging, and pathology to recognize this aggressive malignancy promptly.

## Introduction

Primary small cell carcinoma of the vagina (SmCCV) is an exceptionally rare malignancy, accounting for less than 2% of all gynecologic cancers [1]. Since its first description in 1984, only 44 cases have been reported worldwide [2,3]. SmCCV usually presents in postmenopausal women (mean age approximately 55 years, range 32-81), and postmenopausal bleeding is the most common symptom, although patients may also present with pelvic pain, vaginal mass, or nonspecific local symptoms [4,5].

Histologically, SmCCV closely resembles pulmonary small cell carcinoma [6], which can pose diagnostic challenges, particularly in small biopsies or in distinguishing primary vaginal tumors from metastatic disease. Immunohistochemistry (IHC) is essential for diagnosis. Neuroendocrine differentiation is confirmed by synaptophysin, chromogranin, and insulinoma-associated protein 1 (INSM1). Cytokeratin 20 (CK20) negativity helps exclude Merkel cell carcinoma and colorectal metastases [7,8]. Overexpression of p16 and high-risk human papillomavirus (HPV), particularly HPV-18, has also been documented in some cases [9].

SmCCV carries a poor prognosis, with a reported median overall survival of about 12 months [1,3]. Distant metastases commonly involve the lungs, liver, bone, and lymph nodes. Treatment strategies are largely based on pulmonary small cell carcinoma [6], with platinum-etoposide chemotherapy serving as the standard approach. The use of immune checkpoint inhibitors is emerging but remains insufficiently defined [8,10].

## Case presentation

A 70-year-old postmenopausal woman with a history of hypertension, hyperlipidemia, obstructive sleep apnea, hypothyroidism, and remote cervical carcinoma in situ treated with hysterectomy and bilateral salpingo-oophorectomy in 1986 presented with a three-week history of new-onset vaginal spotting and local discomfort. She denied weight loss, abdominal pain, or changes in bowel or urinary habits.

Pelvic examination revealed a firm, irregular, malignant-appearing exophytic mass measuring approximately 5 cm along the distal vaginal wall at the 7-8 o’clock position near the vulvar margin. The mass demonstrated limited mobility. A palpable right inguinal lymph node was also identified. Given the patient’s history and the aggressive clinical presentation, the initial differential diagnosis included recurrent gynecologic malignancy, primary vaginal carcinoma, metastatic disease, and a high-grade neuroendocrine neoplasm.

The patient was subsequently hospitalized following disease progression and systemic decline. Laboratory evaluation at the time of admission demonstrated normocytic anemia (hemoglobin 9.0 g/dL, hematocrit 27%), thrombocytopenia (platelet count 61 ×10⁹/L), and a normal leukocyte count (8.9 ×10⁹/L). Serum chemistry revealed hyponatremia (sodium 125 mmol/L), acute kidney injury (blood urea nitrogen 44 mg/dL, creatinine 3.0 mg/dL; baseline creatinine approximately 1.0 mg/dL), and elevated lactic acid (3.9 mmol/L). Liver function tests showed transaminitis and cholestatic abnormalities, including elevated aspartate aminotransferase (219 U/L), alanine aminotransferase (39 U/L), alkaline phosphatase (131 U/L), total bilirubin (3.4 mg/dL), and direct bilirubin (3.3 mg/dL), consistent with hepatic dysfunction in the setting of known liver metastases and cirrhosis. Tumor markers were not obtained.

**Figure 1:**
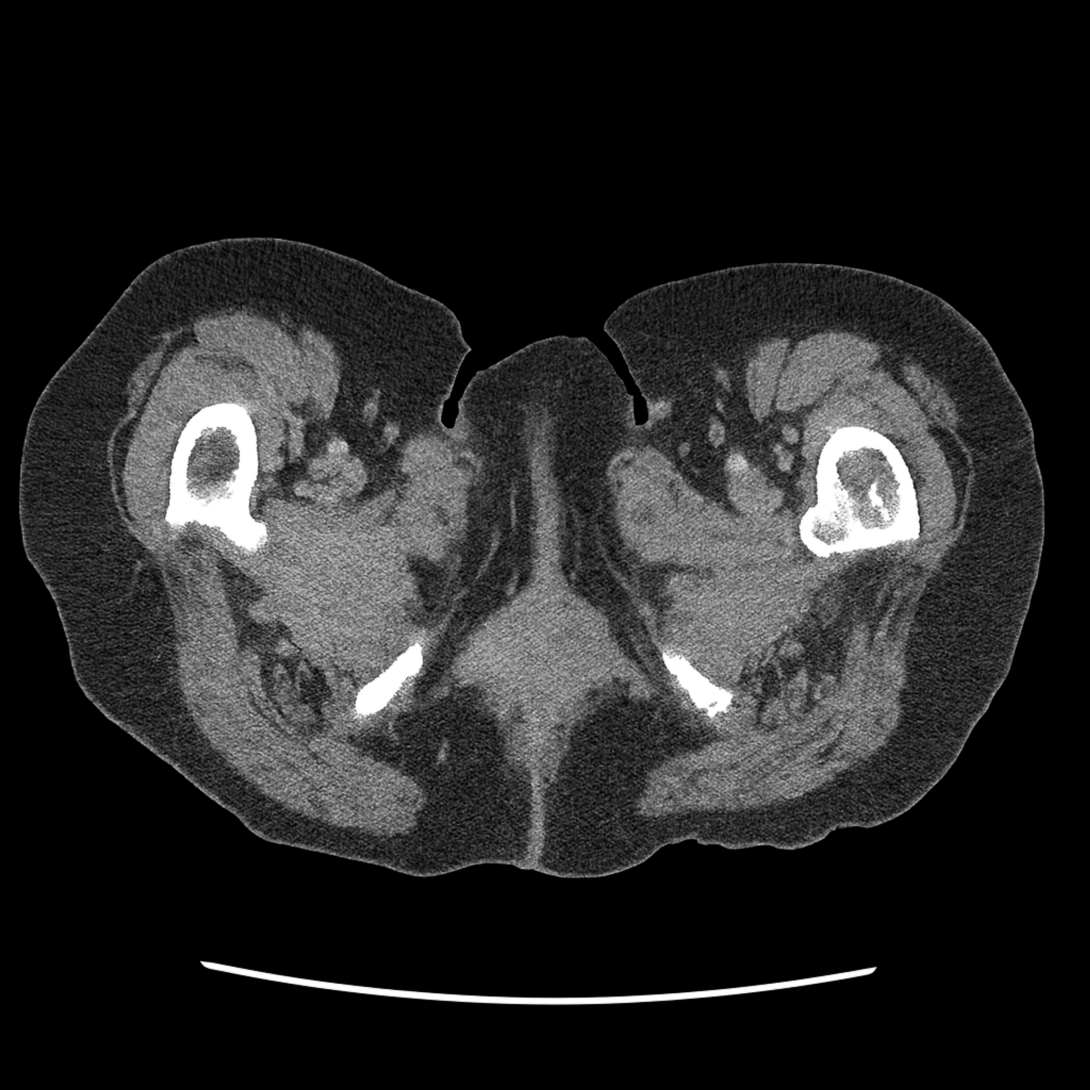
Contrast-enhanced CT abdomen and pelvis demonstrating a hyperdense mass arising from the right lateral vaginal wall.

A PET/CT scan performed on August 8, 2025, revealed an intensely hypermetabolic vaginal wall mass measuring 3.7 × 3.5 cm, with multiple hypermetabolic lesions in the liver and hypermetabolic right external iliac, right inguinal, and mesenteric lymph nodes, as well as a hypermetabolic osseous lesion in the proximal left femoral diaphysis. A corresponding computed tomography (CT) scan of the abdomen and pelvis demonstrating the vaginal mass is shown in Figure 1. MRI of the brain performed one week later showed no intracranial metastases.

**Figure 2:**
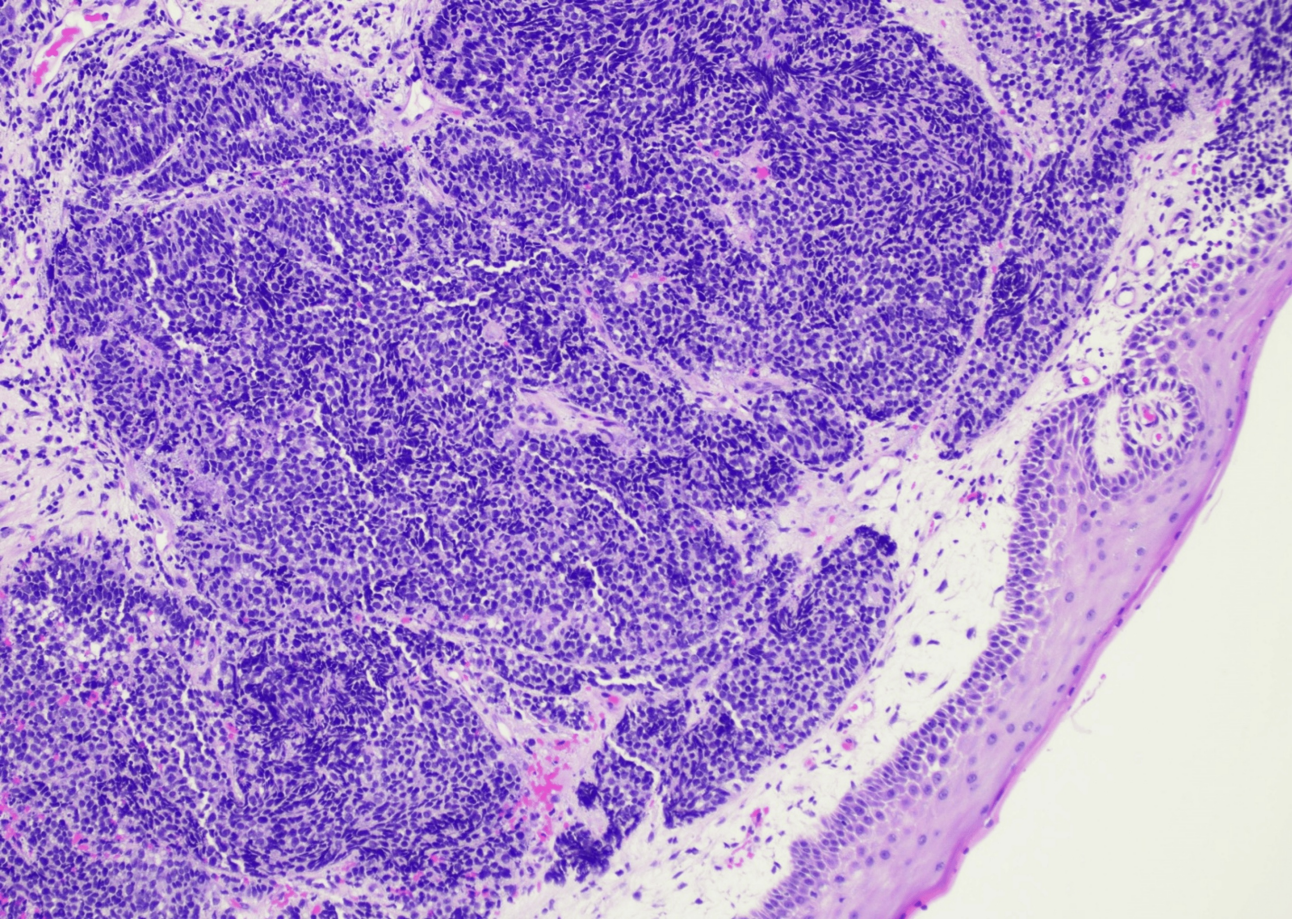
Low-power photomicrograph (H&E, 10×) showing an infiltrative malignant neoplasm composed of sheets and nests of small, round to oval cells beneath the vaginal squamous mucosa.

**Figure 3:**
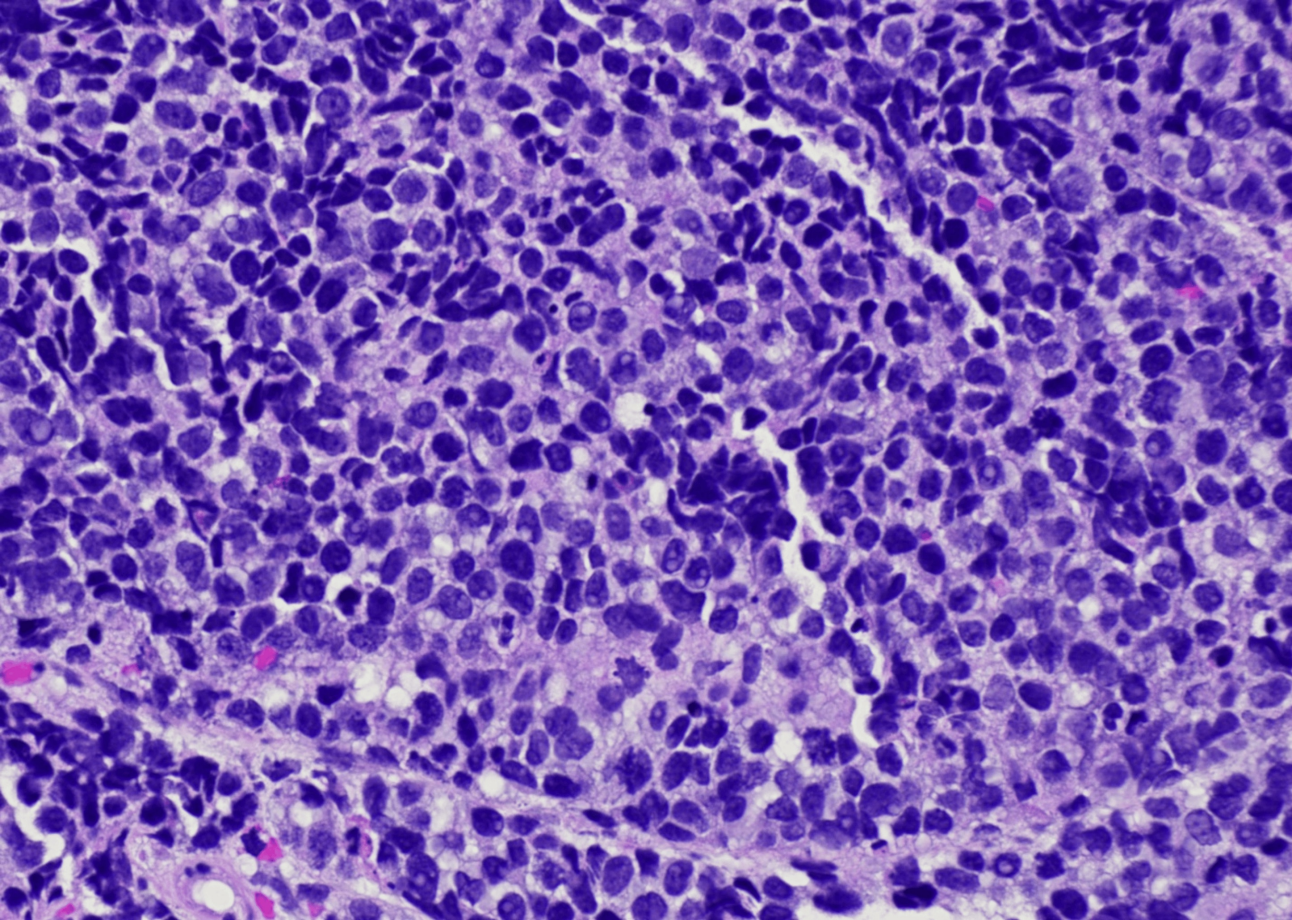
High-power photomicrograph (H&E, 40×) demonstrating tumor cells with scant cytoplasm, hyperchromatic nuclei, nuclear molding, and brisk mitotic activity, consistent with small cell carcinoma morphology.

**Figure 4:**
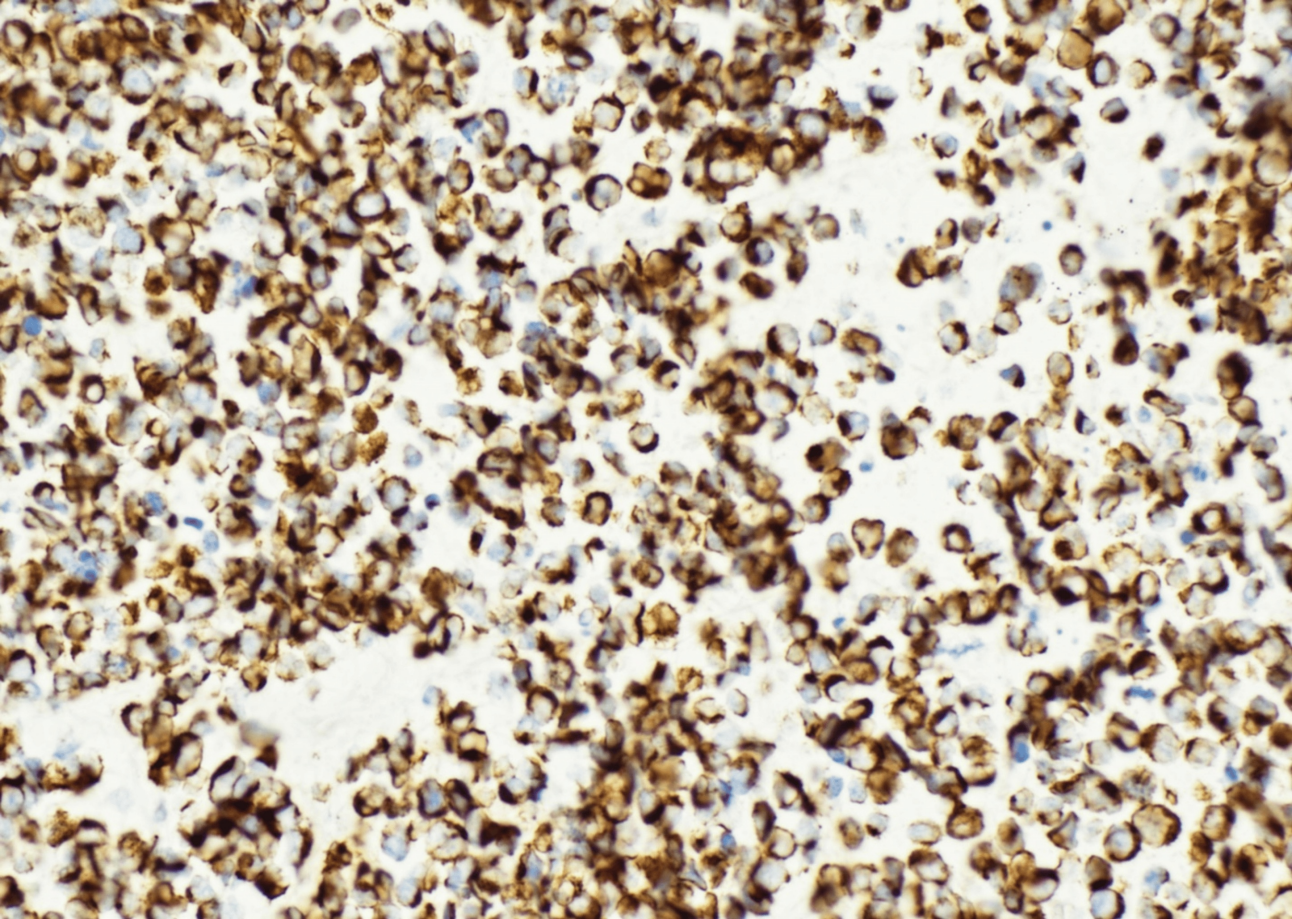
Immunohistochemistry (CAM5.2, 40×) highlighting diffuse cytoplasmic positivity, confirming epithelial differentiation.

**Figure 5:**
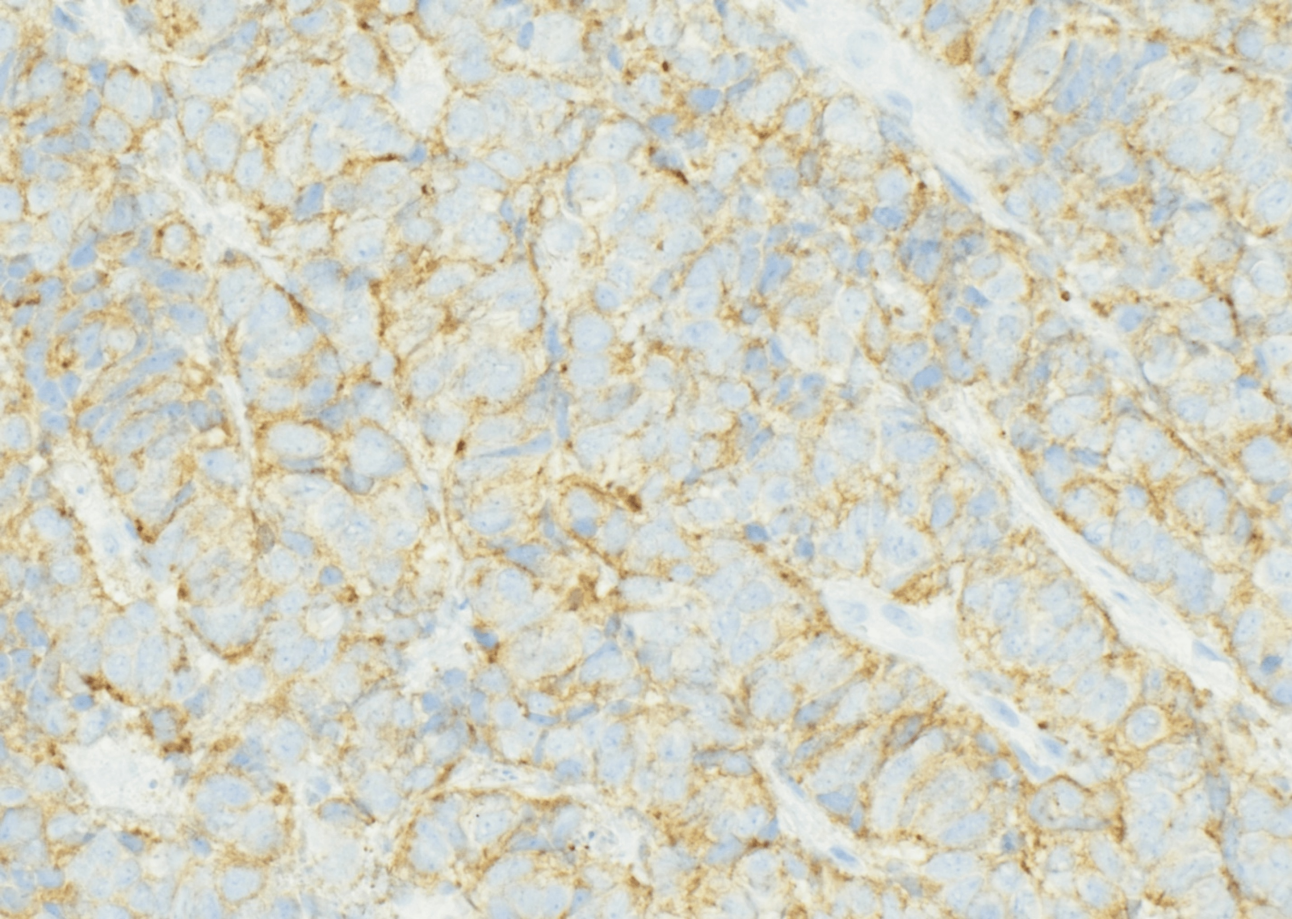
Immunohistochemistry (Synaptophysin, 40×) showing diffuse granular cytoplasmic positivity, supporting neuroendocrine differentiation.

**Figure 6:**
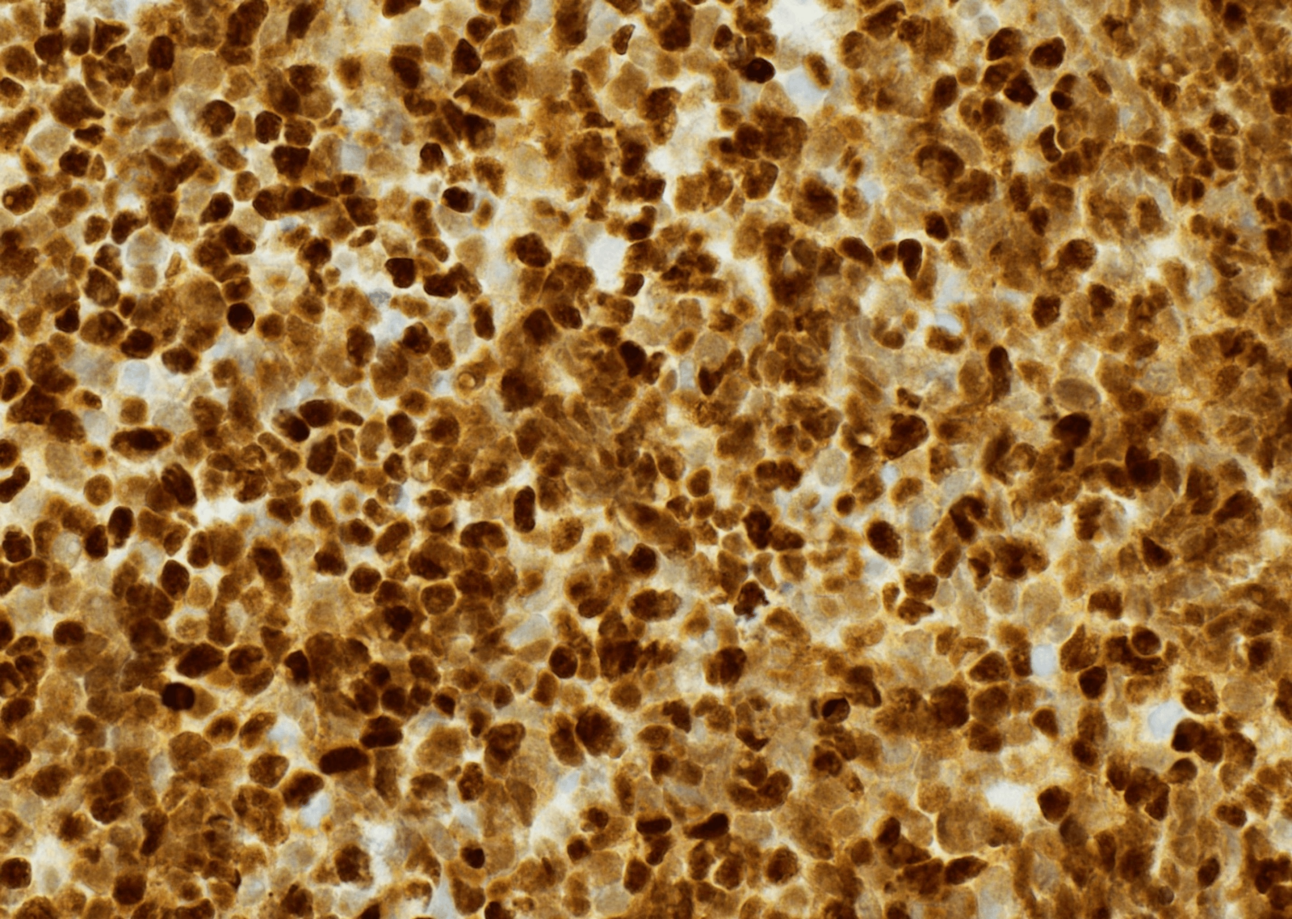
Immunohistochemistry (INSM1, 40×) demonstrating strong nuclear positivity in tumor cells, further confirming neuroendocrine lineage.

**Figure 7:**
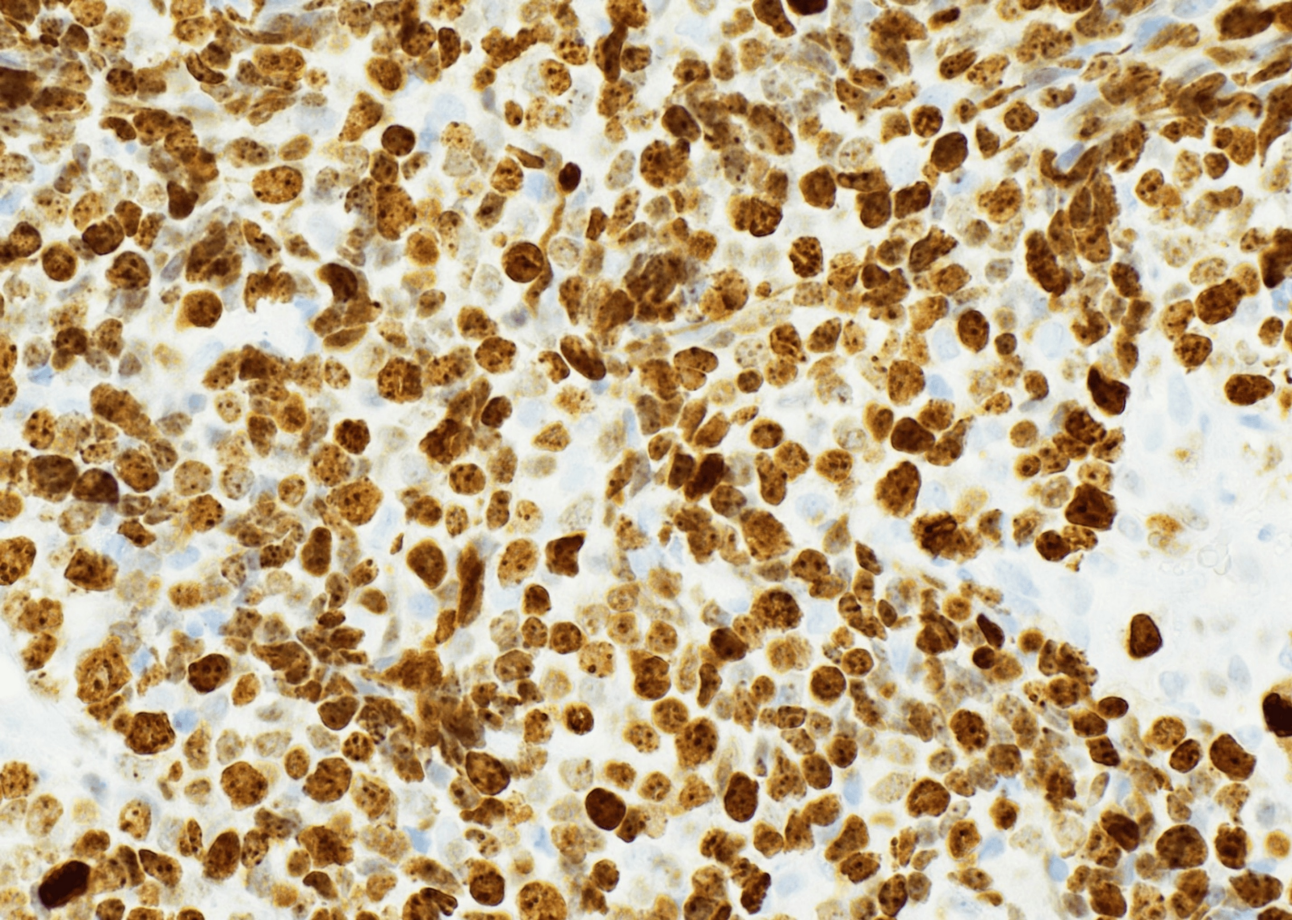
Immunohistochemistry (Ki-67, 40×) showing a markedly elevated proliferation index (>70%), consistent with high-grade small cell carcinoma.

A biopsy of the vaginal mass was obtained. Histologic evaluation showed a densely cellular malignant neoplasm composed of small round blue cells arranged in sheets and nests beneath squamous mucosa (Figures 2-3). The tumor cells exhibited scant cytoplasm, hyperchromatic nuclei, nuclear molding, brisk mitotic activity, and foci of necrosis. Immunohistochemistry (IHC) demonstrated diffuse CAM5.2, synaptophysin, and INSM1 positivity, along with strong p16 expression (Figures 4-6). The Ki-67 proliferation index exceeded 90% (Figure 7). CK20 was negative. These findings supported a diagnosis of high-grade small cell neuroendocrine carcinoma.

Based on the combined histopathologic, immunophenotypic, and radiologic findings, the patient was diagnosed with stage IVB primary small cell neuroendocrine carcinoma of the vagina, according to the International Federation of Gynecology and Obstetrics staging system (FIGO), given the presence of distant metastatic disease involving the liver, bone, and lymph nodes.

Following initiation of systemic therapy, the patient completed two cycles and initially reported improved pain control with preserved functional status (ECOG performance status of 1). However, she subsequently experienced rapid clinical deterioration and was admitted with sepsis, acute kidney injury, hepatic dysfunction, and altered mental status in the setting of extensive metastatic disease. After discussions with the family, goals of care were transitioned to comfort-focused measures. The patient passed away on January 6, 2026.

## Discussion

SmCCV is an extremely rare malignancy, with fewer than 50 reported cases in the literature [1-3]. Because of its rarity, diagnosis requires careful correlation of morphology, immunohistochemistry (IHC), and imaging. Histologically, SmCCV closely resembles pulmonary small cell carcinoma, and the IHC profile often parallels that pattern [6,9]. In this case, diffuse positivity for neuroendocrine markers (INSM1 and synaptophysin) and epithelial marker CAM5.2 supported neuroendocrine differentiation, while CK20 negativity helped exclude Merkel cell carcinoma and colorectal metastases [9,10]. Strong p16 expression may indicate possible high-risk HPV involvement, a feature that has been documented in some cases [8].

To contextualize this case within the published literature, Table 1 provides a comparative summary of key reported cases and series of SmCCV, including patient characteristics, diagnostic features, treatments, and outcomes.

**Table 1:** Comparative summary of reported cases of primary small cell carcinoma of the vagina. Comparative summary of published primary cases of small cell carcinoma of the vagina, including clinical presentation, pathologic findings, treatment, and outcomes. Only original case reports and case series are included. Clinical details were cross-referenced with the systematic review by Capote et al. (2023) [2] for completeness but were derived from the original publications listed.

Study (Year)	No. of Cases	Age at Diagnosis	Stage at Diagnosis	Metastatic Sites	Key IHC / Pathology	Treatment	Outcome
Ribeiro-Silva et al., 2003 [9]	1	45	Localized	None	Chromogranin A+, synaptophysin+	Surgery + radiotherapy	Alive at 6 months
Tamura et al., 2013 [6]	1	81	Localized	None	Neuroendocrine markers+	Surgery ± radiotherapy	NR
Oliveira et al., 2013 [4]	1	Postmenopausal	Advanced	Metastatic disease reported	Neuroendocrine markers+	Multimodal therapy	Poor prognosis
Zhang et al., 2021 [1]	2	Adults	Early	None	Small cell morphology	Surgery ± adjuvant therapy	One DFS at 36 months
Puja & Arya, 2021 [3]	1	NR	NR	NR	Small cell morphology	Platinum-based chemotherapy + RT	NR
Pongsuvareeyakul et al., 2022 [8]	1	54	Advanced	LN	INSM1+, synaptophysin+, discordant HPV	Chemoradiation	Short follow-up
Present case (2025)	1	70	IVB	Liver, bone, LN	INSM1+, synaptophysin+, CAM5.2+, p16+, CK20−	Carboplatin + etoposide + atezolizumab	Early follow-up

SmCCV is associated with an aggressive clinical course. Reported median overall survival is approximately 12 months [1,3], and many patients present with advanced disease. The most frequent metastatic sites include the liver, bone, and lungs [4,5]. The patient described here presented at an older age than typically reported and had widespread metastatic disease, classified as FIGO (International Federation of Gynecology and Obstetrics) Stage IVB at diagnosis.

As summarized in Table 2, FIGO stage at diagnosis is strongly associated with survival outcomes, with early-stage disease demonstrating a more favorable prognosis compared with advanced-stage disease, based on data derived from the systematic review by Capote et al. (2023) [2]. Beyond FIGO stage, additional factors influencing survival include a high Ki-67 proliferation index reflecting aggressive tumor biology, larger primary tumor size, human papillomavirus-associated status (often inferred by strong p16 expression), and advanced patient age, all of which have been associated with poorer outcomes in reported cases of vaginal small cell neuroendocrine carcinoma.

**Table 2:** Stage distribution and survival outcomes in small cell carcinoma of the vagina (Capote et al., 2023 [2]) Distribution of FIGO stage at diagnosis and corresponding median overall survival among reported cases of primary small cell carcinoma of the vagina. The data are summarized and derived from the systematic review by Capote et al. (2023) [2] and are not reproduced verbatim from the original publication. The table highlights the association between disease stage at diagnosis and overall survival.

FIGO Stage	Proportion of Cases (%)	Median Overall Survival (months)	Key Notes
Stage I	~20%	Not reached	Most favorable prognosis
Stage II	~25%	12	Intermediate prognosis
Stage III	~20%	12	Parametrial involvement
Stage IVA	~10%	9	Adjacent organ invasion
Stage IVB	~25%	8	Distant metastatic disease
Overall	—	12	Platinum-based therapy is associated with improved survival

There are no dedicated treatment guidelines for SmCCV due to its rarity. Management is generally extrapolated from pulmonary small cell carcinoma, for which platinum-etoposide chemotherapy remains the standard approach [7,10]. In recent years, the incorporation of immunotherapy has shown benefit in extensive-stage small cell lung cancer, most notably in the IMpower133 trial, where the addition of atezolizumab improved overall survival [7]. Although evidence for immunotherapy in extrapulmonary small cell carcinomas is limited, similar regimens are increasingly used in clinical practice [10]. The treatment strategy used in this case aligns with these evolving approaches.

This report highlights several notable features: an older age at presentation, widespread metastatic disease at diagnosis, use of INSM1 in the diagnostic IHC panel, and incorporation of chemoimmunotherapy as initial treatment. These details contribute to the limited existing literature on SmCCV.

The primary limitation of this report is the short follow-up. At the time of writing, the patient has completed two cycles of systemic therapy, and radiologic response has not yet been assessed. Continued follow-up will be necessary to determine treatment effectiveness and disease trajectory.

## Conclusions

SmCCV is an exceptionally rare and aggressive malignancy with limited evidence to guide diagnosis and management. Accurate identification requires integration of histopathology, immunohistochemistry (IHC), and radiologic exclusion of more common primary sites. This case highlights two important considerations. First, diagnostic rigor is essential; the use of comprehensive IHC panels, including newer neuroendocrine markers such as INSM1, helps confirm lineage and exclude morphologic mimics. Second, management remains challenging. Although the prognosis for advanced-stage SmCCV is poor, the emerging use of immune checkpoint inhibitors in small cell lung cancer has prompted interest in applying similar strategies to extrapulmonary small cell carcinomas.

Clinicians should consider SmCCV in the differential diagnosis of atypical vaginal masses, particularly in older women. Continued case reporting and collaborative research are necessary to better define optimal therapeutic approaches and to clarify whether immunotherapy may offer benefit in this rare tumor type.
